# Does integration with national registers improve the data completeness of local COVID-19 contact tracing tools? A register-based study in Norway, May 2020 - September 2021

**DOI:** 10.1186/s12913-023-10540-5

**Published:** 2024-01-17

**Authors:** Hinta Meijerink, Mohamed Shelil, Jagrati Jani-Bølstad, Evy Therese Dvergsdal, Elisabeth Henie Madslien, Madeleine Wilberg, Ragnhild Bassøe Gundersen, Johan Ivar Sæbø, Anne Asmyr Thorseng, Bjørn Gunnar Iversen

**Affiliations:** 1 Department of Infection Control and Vaccines, Norwegian Institute of Public Health, Oslo, Norway; 2 Department of Infection Control and Preparedness, Norwegian Institute of Public Health, Oslo, Norway; 3 Tromsø municipality, Tromsø, Norway; 4 Department of Infectious Disease Registries, Norwegian Institute of Public Health, Oslo, Norway; 5 University of Oslo, Oslo, Norway

**Keywords:** COVID-19, Contact tracing, Public health surveillance, Database management systems, Systems integration, Disease outbreaks, Norway

## Abstract

**Background:**

During the COVID-19 response in Norway, many municipalities used the Fiks contact tracing tool (FiksCT) to register positive individuals and follow-up contacts. This tool is based on DHIS2, an open source, web-based platform. In this study we examined if data completeness in FiksCT improved after integration with national registers between May 2020 and September 2021.

**Methods:**

Data from municipalities using FiksCT was extracted from the Norwegian Emergency Preparedness Register for COVID-19 (Beredt C19). We linked FiksCT data to the Norwegian Surveillance System for Communicable Diseases (MSIS), the National Population Register (FREG), and the Norwegian Vaccine Registry (SYSVAK) using unique identification numbers (ID). Completeness for each variable linked with a national register was calculated before and after integration with these registers.

**Results:**

Of the 125 municipalities using FiksCT, 87 (69.6%) agreed to share and upload their data to Beredt C19. Data completeness for positive individuals improved after integration with national registers. After integration with FREG, the proportion of missing values decreased from 12.5 to 1.6% for ID, from 4.5 to 0.9% for sex, and from 1.2 to 0.4% for date of birth. Missing values for vaccine type decreased from 63.0 to 15.2% and 39.3–36.7% for first and second dose, respectively. In addition, direct reporting from FiksCT to MSIS increased the proportion of complete records in MSIS (on the selected variables) from 68.6% before to 77.0% after integration.

**Conclusion:**

The completeness of local contact tracing data can be improved by enabling integration with established national registers. In addition, providing the option to submit local data to the national registers could ease workload and reduce the need to collect duplicate data.

**Supplementary Information:**

The online version contains supplementary material available at 10.1186/s12913-023-10540-5.

## Background

Since the start of the COVID-19 pandemic in 2020, one of the main strategies for disease control in Norway (population of 5.5 million) has been testing individuals, isolating those who were infected, and tracing and quarantining close contacts [1, 2]. Health teams under each municipality were responsible for identifying cases, initiating and executing contact tracing as well as entering data and reporting to the national data registers [3]. Initially, the lack of efficient digital tools for contact tracing was challenging, but by the second half of 2020 digital systems were implemented to fit the complex COVID-19 response [4–6]. One such system was based on DHIS2, an open source, web-based platform developed by the University of Oslo that has been used worldwide [5–8]. On behalf of the municipalities, the Norwegian Association of Local and Regional Authorities (KS) deployed and managed a DHIS2 based contact tracing tool named FiksCT. This tool was integrated into a centralized platform (called Fiks), which provides common digital solutions for municipalities as well as facilitates information sharing between national registers and provides secure data storage for the municipalities. FiksCT was launched in May 2020 and about one third of the 357 Norwegian municipalities used FiksCT for contact tracing during the SARS-CoV-2 pandemic. New functionalities were continuously developed, including integration with the national registers.

**Fig. 1:**
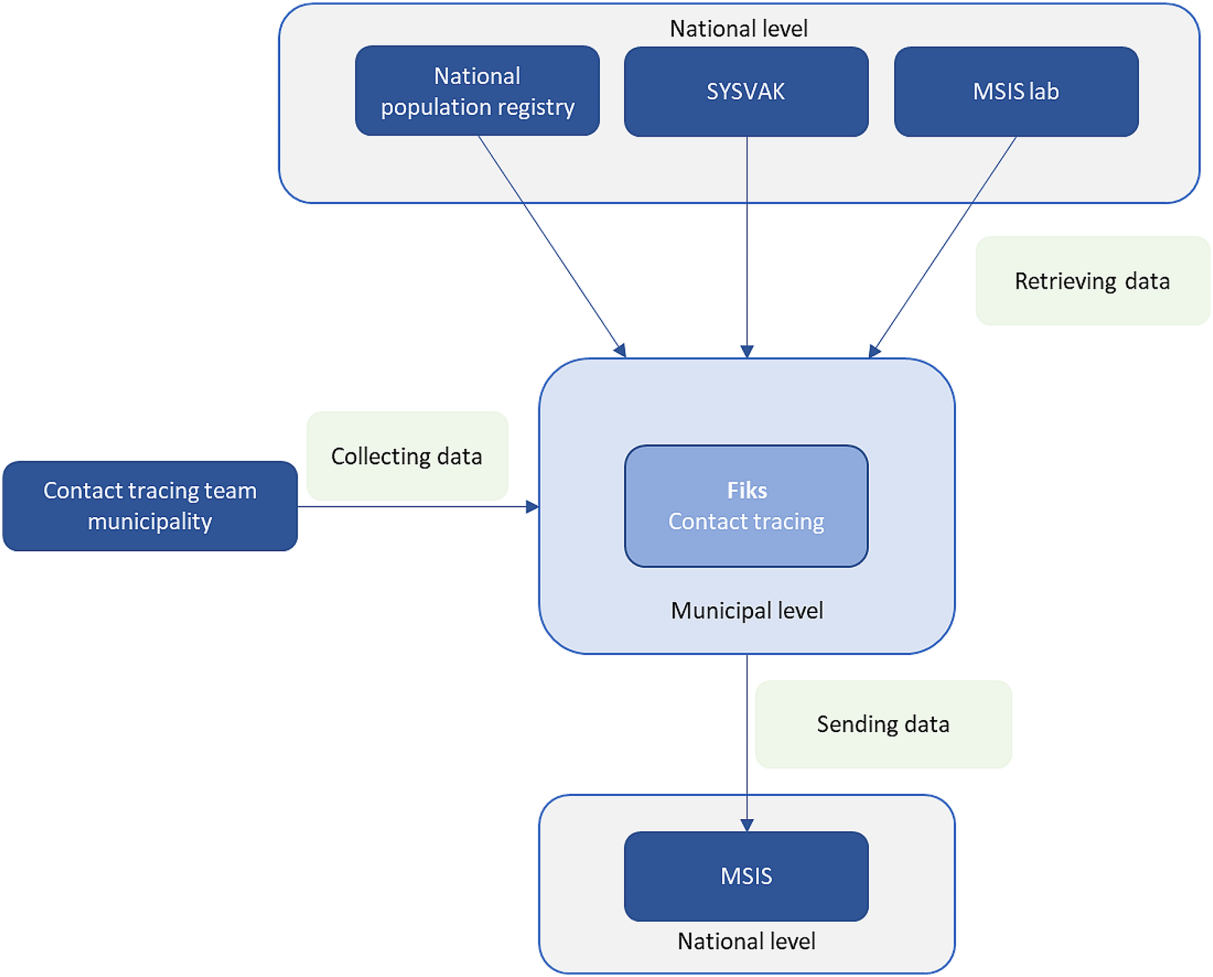
Overview of the FIKS contact tracing (FiksCT) tool, based on DHIS2 platform, and integration with national registers. SYSVAK: Norwegian Immunisation Registry; MSIS: Norwegian Surveillance System for Communicable Diseases; MSIS lab: laboratory database in MSIS; FIKS: digital solution for data collection at municipal level. Dark blue boxes: data outside the FiksCT. Light blue boxes: data in FiksCT

By law, medical microbiological laboratories and clinicians report all cases of COVID-19 to the Norwegian Surveillance System for Communicable Diseases (MSIS) with full patient identification [9]. To facilitate case reporting from municipalities to MSIS, integration between FiksCT and MSIS was developed. This integration allowed for an automated real-time reporting system. Other integrations between the FiksCT and national registers, such as the National Population Register (FREG), the Norwegian Immunization Registry (SYSVAK) and MSIS laboratory database (MSIS-lab) (Fig. 1) allowed restricted information access [10–12]. Linkage between health registers is not routinely performed and was previously only permissible under specific circumstances.

FiksCT was a secure system where superusers from the municipalities had control over access, and data quality control. Data access was restricted to authorized personnel, e.g. personnel trained in contact tracing. These individuals were granted two-level security to login to the system by using their personal identification number. Users were only able to access data from their own municipality. Even though contact tracing was coordinated differently in each municipality, the FiksCT was standardized and structured in shaping the content of the data. All the contact tracers were employed in each municipality under the responsibility of the respective municipal doctor. Data from each case or contact was entered using unique Norwegian national identity number for both the contract tracer and the target individuals. The use of unique identity number allowed retrieval of data from other systems. None of the variables in this tool were mandatory, allowing flexibility in the recording system as well as local autonomy on which data were important to record. The aim of this study was to assess whether the completeness of contact tracing data in FiksCT at the municipal level as well as in MSIS at the national level improved after integration between FiksCT and national registers. This paper describes data completeness in the FiksCT and the impact of integration of registers on completeness of contact tracing data and MSIS data in Norway between May 2020 and September 2021.

## Methods

### Study design and data sources

This study was a retrospective cohort study using register-based data in Norway from 15 May 2020 until 27 September 2021. We extracted data from the Emergency Preparedness Register for COVID-19 (Beredt C19) [13], which is located on a secure server at Norwegian Institute of Public Health (NIPH). This register contained various national and local registers which are collected routinely and regularly updated. For this study, we included data from SARS-CoV-2 positive cases and their close contact reported through FiksCT from all municipalities that agreed to share FiksCT data with NIPH via Beredt C19. In addition, we included data from the national registers FREG, SYSVAK, MSIS and the MSIS laboratory for the same municipalities (see additional file, table S1). FREG is the National Population Register and contains information on everyone that resides or has resided in Norway [10]. SYSVAK, MSIS and MSIS laboratory are national health registers that cover mandatory reporting of notifiable diseases and vaccinations provided in the national vaccine programmes [9, 11, 12]. Systematic, regular quality control of these registers is mandated by law. We used anonymized individuals’ identification numbers in BeredtC19 to link individual data from the above-mentioned registers [14].

### Study population and study period

We included all positive cases recorded in FiksCT in Norway from 15 May 2020 until 27 September 2021 (see additional file, table S2). This period was selected based on the start of FiksCT data collection until the time at which contract tracing was no longer part of the mandatory COVID-19 response in Norway. For duplicate positive cases registered less than 60 days apart, we excluded the least complete record. An index wa defined as a record from a specific infection of an individual; re-infections would thus result in two indexes for one person. For contacts, multiple linkages to the same index were considered duplicates.

### Data analyses

The variables included in this study were all variables in FiksCT that were available in the national register Beredt C19. Completeness for each individual variable in the index and close contact modules in FiksCT was calculated as the proportion of records with values. Binomial “exact” calculation was used to estimate 95% confidence intervals (95%CI) for proportions. We compared the completeness of relevant variables before and after integration with other national registers using date of integration for the specific municipality. Integration with SYSVAK was analysed after start of vaccination in Norway, 1 January 2021. In addition, we evaluated the completeness of clinical data in the national MSIS database, before and after integration with FiksCT. All analyses were performed using STATA/SE 16.0 [15].

## Results

Of the 125 municipalities using FiksCT, 87 (69.6%) agreed to share and upload their data to Beredt C19 (additional file 1: table S2) and were included in our analyses. Overall, at the end of the study period, 46% of municipalities used all integration modules, 46% use some, and 8% only use basic FiksCT (additional file 1: table S3).

From 19 May 2020 to 27 September 2021, 57 496 positive cases were registered in FiksCT, while 276 duplicate records were excluded (0.5%). Of the remaining records, 197 (0.3%) had multiple entries (> 60 days apart; presumed reinfections). The number of cases recorded per municipalities ranged from one to 11 382 (supplement table S2). Of the 56 614 positive cases, 9 119 (16.1%) had no registered contacts. Of the 183 198 contacts registered in FiksCT, we excluded 1 149 duplicates (0.6%) and 26 222 (14.3%) had multiple entries (contacts to different positive cases).

Between 19 May 2020 and 27 September 2021, a total of 179 016 COVID-19 cases were reported in Norway and this study covers 31,6% (56 614) of these cases. The municipalities included in this study reported 65 300 cases during the same period, and this study covers 86.7% of these individuals [16].

### Completeness FiksCT

Of the 56 614 positive cases included, only 1 584 (2.8%) did not have a national identification number, while the completeness of other variables varied from 22.2% (civil status) to 99.5% (age) (additional file 1: table S4). Of the close contacts, 30 906 (17%) did not have a national identification number and 53 100 (29%) did not have a reference number to link with an index case. Overall, data completeness was higher in larger municipalities compared to smaller municipalities (additional file 1: table S5).

### Integration with national registers

The completeness of variables included in the integration with the Norwegian Population Register improved for identity number, sex, and date of birth (Table 1). The data completeness of vaccine status improved after integration with SYSVAK; the proportion of missing vaccine type decreased from 63 to 15% and date from 6.2 to 2.6% after first dose (Table 1). Within the study period, 65 300 positive cases from included municipalities were reported to MSIS. The proportion of complete records in MSIS (on included variables) increased from 68.7% (95%CI: 68.0–69.5%) before to 76.9% (95%CI: 76.0-77.7%) after integration. Overall, the completeness for most variables in MSIS improved after municipalities included the integration function in FiksCT (Table 1), except for reason for testing and place/type of exposure.

**Table 1:** Overview of the missing values for variables in FiksCT that were linked with national registers before and after date of integration at the municipality

Variable	Missing values			
	Before integration		After integration	
	*Missing*	*%*	*Missing*	*%*
**Data in municipal FiksCT based on linkage with Norwegian Population Register** ^#^				
	(n = 6420)		(n = 50 190)	
ID	802	12.5	782	1.6
Sex	289	4.5	436	0.9
Date of birth	75	1.2	181	0.4
**Data in municipal FiksCT based on linkage with SYSVAK** ^#^				
	(n = 38 897)		(n = 6 197)	
Type of first vaccine	24 344	62.6	917	14.8
Date of first vaccine	559	6.2	78	2.6
Type of 2nd vaccine	3 617	39.3	1 111	36.7
Date of 2nd vaccine	270	5.6	24	1.7
Data in national MSIS register including variable submitted through FiksCT*				
	(n = 43 844)		(n = 21 456)	
Date of symptoms	16,051	36.6	7 524	35.1
Home care admission	20 537	46.8	3 980	18.6
Close contact	9 239	21.1	4 221	19.7
Infected outside Norway	14 034	32.0	5 722	26.7
Reason for test	7 358	16.8	4 880	22.7
Underlying conditions	13 126	29.9	5 006	23.3
Place/type of exposure	8602	19.6	4 773	22.3

SYSVAK: Norwegian Immunisation Registry, MSIS: Norwegian Surveillance System for Communicable Diseases. ^#^Missing values for variables in FiksCT. ^*^Missing values of variables in MSIS, for variables included in the FiksCT

## Discussion

### Main finding

This study demonstrated that the completeness of local contact tracing data can be considerably improved by allowing integration with existing national register data. Retrieving data from the three quality-assured national databases as well as the ability to report real-time data directly from the local FiksCT tool to the national registers, eases the workload for the contact tracers and reduces the need to collect duplicate data.

### Interpretation of results

The COVID-19 pandemic revealed pitfalls in preparedness plans and underscored a need for better integrated surveillance systems for quicker response [6, 8]. Even though this study is country-specific, the main results are generalisable; information sharing between registers, thus requiring less data entry in surveillance systems, will contribute to more efficient response. The importance of integrated surveillance tools to allow quicker public health response has also been reported by others and can be relevant for other aspects of public health surveillance and response [17–20]. In addition to being an important tool to trace and identify cases and contacts at the local level, contact tracing data represents a unique source of information that can be used by national health authorities to provide essential knowledge about disease dynamics, such as the transmissibility across different subgroups, potential differences between virus variants and the effect of interventions [21]. This information is critical in understanding basic epidemiological patterns and how to combat a new evolving disease. Ideally, common tools for contact tracing should be available within the country to allow harmonised data collection from all regions to enable more efficient collaboration between regions as well as better comparability at national level.

We have previously reported that other types of digital tools, namely those using Bluetooth technology to identify close contacts, can be useful in identifying close proximity of “unknown” positive cases [22, 23]. However, many have reported that those at highest risk of infection after contact with a known case are household members and others with prolonged close contact [22, 23], but this can be dependent on the virus variant [24]. Therefore, FiksCT and other contact tracing tools may play a large role when responding to outbreaks to identify and follow those at highest risk of infection. Various papers have reported the effect of non-pharmaceutical interventions, including contact tracing, but the role of digital tools for contact tracing are not specially addressed [25–28]. Many countries, like Norway, have mandatory reporting to a national surveillance system for specific infectious diseases. Depending on the organisation of a public health event response, these systems may not be suitable to collect contact tracing data. It is therefore important to have alternative flexible tools for response that can be linked or integrated with these national surveillance systems.

### Limitations and strengths

Due to data privacy and protection agreements, we could not assess all variables included in FiksCT and the data quality reported in this study may not be representative for the other variables in FiksCT [14]. The study has limitations related to the development and use of FiksCT data for research and surveillance. General guidelines and training in the use of the system varied depending on factors, such as the case load and available human resources. During the pandemic response, the demand for human resources was high, resulting in fast recruitment and task shifting to cover the needs of contact tracing. Furthermore, FiksCT was rapidly developed and continuously changed to adjust to a dynamic evolvement of epidemiological situations. This contributed to many changes in contact tracing strategies and practices over time. As integration with national registers also happened over time, some of the improved completeness could be associated with improved data collection or increased coverage of vaccines resulting in higher completeness.

## Conclusion

In conclusion, the data completeness of the digital contact tracing tool improved by connecting information from national registers. Overall, identifying and developing methods and tools to improve efficiency of data collection during contact tracing is important for a timely response. This is especially crucial during outbreaks spanning large regions and lasting an extended period with multiple stakeholders involved. Evaluating digital tools for contact tracing, such as FiksCT, is essential to refine and improve these tools for outbreak responses in the future. Flexible systems are important for efficient response to unexpected events, such as pandemics, and these should build upon and utilize data from established, quality-ensured, national data registers to allow quick monitoring. This study highlights the importance for countries with high-quality registers and the ability to link these using personal identification numbers, to prepare a legal basis and a system for connecting databases. This will enable rapid data analysis and response both during emergencies and in peace time, while at the same time upholding data security and GDPR. A contact tracing tool, such as FiksCT, can feed into such a set of relational databases and assist the contact tracers in their work.

### Electronic supplementary material

Below is the link to the electronic supplementary material.

Supplementary Material 1: Data used and results from additional analyses

## Data Availability

The datasets analysed for this study come from the national Emergency preparedness register for COVID-19 (Beredt C19), housed at the Norwegian Institute of Public Health. This register comprises data from a variety of national registers and legal restrictions prevent the researchers from sharing the dataset used in the study. However, external researchers can request access to linked data from the same registers from outside the structure of Beredt C19, as per normal procedure for conducting health research on register data in Norway (https://www.helsedata.no). We understand that BMC encourage all authors to share their data. Unfortunately we are not able to do so due to data protection and related laws. However, anyone interested in using the same data, they can apply through the official channels as indicated via https://www.helsedata.no. Further information on the Emergency preparedness register for COVID-19, including access to data from each data source, is available at:https://www.fhi.no/en/id/corona/coronavirus/emergency-preparedness-register-for-covid-19/.
