# Supplementary material for: Does integration with national registers improve the data completeness of local COVID-19 contact tracing tools? A register-based study in Norway, May 2020 - September 2021
Source: BMC Health Serv Res. 2024 Jan 17;24:96. doi: 10.1186/s12913-023-10540-5 (PMC10795336; doi:10.1186/s12913-023-10540-5)
Supplement: Supplementary file 1 — Supplementary Material 1: Data used and results from additional analyses [file 12913_2023_10540_MOESM1_ESM.pdf]

Additional file for manuscript

“Does integration with national registries improve the data completeness of local COVID-19 contact tracing tools? A register-based study in Norway, May 2020 - September 2021”

Table of Contents

Overview of registers used 2

Table S1. Overview of registers used for this study. *2*

Overview of municipalities included 2

Table S2. Overview of municipalities using FIKS platform for the registration of SARS-CoV-2 cases, including the number of cases reported Numbers smaller than 5 are reported as ≤5. .... *2*

Integration of FiksCT with national registries 4

Table S3. Overview of the number of municipalities using the FIKS contact tracing tool and integration functionalities within the tool. *4*

Proportion of missing data in FiksCT 5

Table S4. Number of records with missing values for each of the variable included in the Index module of Fiks contact tracing tool, 19 May 2020 to 27 September 2021. .... *5*

Table S5. Number of records with missing values for each of the variable included in the index case module of FIKS contact tracing tool by municipality size, 19 May 2020 to 27 September 2021. .... *5*

Overview of registers used

For this study, we extracted data from the Norwegian emergency preparedness registry for COVID-19 (Beredt C19), which contains data from various national and local registers collected routinely and is located on a secure server at Norwegian Institute of Public Health (NIPH). We included data from FiksCT as well as the national registries FREG, SYSVAK, MSIS and the MSIS laboratory registry and linked these using anonymised unique identifiers. Table S1 gives an overview of the registers used for this study.

Table S1. Overview of registers used for this study.

Register name	Abbreviation	Coverage	Ownership
National Population Register	FREG	National	Norwegian Tax Administration
Norwegian Immunization Registry	SYSVAK	National	Norwegian institute of Public Health
Norwegian Surveillance System for Communicable Diseases	MSIS	National	Norwegian institute of Public Health
Norwegian Surveillance System for Communicable Diseases – laboratory register	MSIS – lab	National	Norwegian institute of Public Health
Fiks contact tracing platform	FiksCT	Regional	The Norwegian Association of Local and Regional Authorities (KS)

Overview of municipalities included

Data was included from municipalities who use the FiksCT for contact tracing and agreed to share their data with NIPH. Of the 125 municipalities using the FIKS platform, 87 (69.6%) agreed to share and upload their data with Beredt C19. Table S2 gives a list of all municipalities included with the total number of positive cases reported.

Table S2. Overview of municipalities using FIKS platform for the registration of SARS-CoV-2 cases, including the number of cases reported Numbers smaller than 5 are reported as ≤5.

Municipality	Positive cases registered (n)
Hattfjelldal kommune	≤5
Lebesby	≤5
Lødingen	≤5
Vardø	≤5
Etnedal	7
Kárásjohka - Karasjok	8
Nordkapp	8
Engerdal	9
Bykle	9
Rollag	11
Evenes	13
Bardu	15
Gamvik	15
Hemnes	15
Tokke	16
Tysnes	22
Øystre Slidre	23
Porsanger - Porsángu - Porsanki	23
Aremark	26
Berlevåg kommune	28
Overhalla	28

Municipality	Positive cases registered (n)
Sør-Aurdal	29
Nore og Uvdal	29
Lom	34
Hareid	35
Stor-Elvdal	46
Aukra	49
Kvåfjord kommune	51
Grong	52
Vefsn	53
Dovre	54
Krødsherad	55
Evje og Hornnes	57
Flesberg	58
Vadsø	62
Målselv	63
Siljan	75
Søndre Land	85
Sigdal	94
Sør-Varanger	96
Herøy (Møre og Romsdal)	100
Nordre Land	108
Oppdal	125
Sortland - Suortá	130
Tynset	131
Volda	148
Namsos	148
Narvik	158
Sel	165
Ulstein	168
Åmot	185
Notodden	186
Midtre Gauldal	186
Nærøysund	188
Sunndal	210
Sula	214
Nome	231
Hole	243
Jevnaker	248
Midt-Telemark	260
Melhus	281
Lunner	286
Lyngdal kommune	307
Alta	321
Verdal	323
Gran	381
Vestre Toten	384
Østre Toten kommune	437
Harstad	454
Elverum	719
Enebakk	741
Øvre Eiker	768
Karmøy	821

Municipality	Positive cases registered (n)
Frogn	883
Lillehammer	914
Vestby	969
Nesodden	1059
Færder	1206
Ringerike kommune	1223
Kongsberg	1263
Nittedal	1320
Tromsø	2915
Lørenskog	3893
Nordre Follo	4882
Asker	5714
Lillestrøm	7872
Bærum	11382

Integration of FiksCT with national registries

To facilitate easy reporting from municipalities to MSIS at a national level, the FIKS platform included the integration of MSIS and allows direct reporting of cases. In addition, other national registries are integrated to allow the FIKS contact tracing system to download information from these registries, including the National Population Register (Folkeregisteret), the Norwegian Immunization Registry (SYSVAK) and MSIS laboratory. In addition, the mandatory clinical reporting to MSIS has also been integrated in FIKS, which allows direct uploading of data. Functionalities of the tool were expanded over time, including the integration of the national registries, and municipalities could choose which functionalities to order against costs. Table S3 gives an overview of the use of this integration by municipalities.

Table S3. Overview of the number of municipalities using the FIKS contact tracing tool and integration functionalities within the tool.

Name of national registry	Number of municipalities (n=80)	Proportion
Norwegian Surveillance System for Communicable Diseases (MSIS)	50	63%
MSIS Laboratory	61	76%
National Population Register	72	90%
Norwegian Immunisation Registry (SYSVAK)	48	60%
**All modules**	**37**	**46%**

Proportion of missing data in FiksCT

Table S4 and Table S5 give an overview of the number of records of positive cases with missing information in FiksCT, overall (S4) and by municipality size (S5).

Table S4. Number of records with missing values for each of the variable included in the Index module of Fiks contact tracing tool, 19 May 2020 to 27 September 2021.

Variable		Missing values			Completeness
		N	n	%	%
National ID number		56,614	1,584	2.8	97.2
Sex		56,614	725	1.3	98.7
Age - any		56,614	256	0.5	99.5
	Age	56,614	306	0.5	99.5
	Date of birth	56,614	256	0.5	99.5
Country of birth		56,614	37,468	66.2	33.8
Civil status		56,614	44,034	77.8	22.2
Profession		56,614	32,555	57.5	42.5
	Profession	56,614	25,139	44.4	55.6
	Profession category	56,614	34,258	60.5	39.5
Vaccination*	Type of first vaccine	45,789	25,925	56.6	43.4
	Date of first vaccine	12,073	643	5.3	94.7
	Type of second vaccine	12,073	4,737	39.2	60.8
	Date of second vaccine	6,305	299	4.7	95.2

Vaccination only included for index recorded in 2021. Dates of first and second vaccine only given for those with a type of vaccine.

Table S5. Number of records with missing values for each of the variable included in the index case module of FIKS contact tracing tool by municipality size, 19 May 2020 to 27 September 2021.

Variable	Missing values					
	**<5000 inhabitants** (n=1,155; 35 municipalities)		**5000 - 14999 inhabitants** (n=6,296; 30 municipalities)		**≥ 15 000 inhabitants** (n=49,163; 22 municipalities)	
	N	%	N	%	N	%
National ID number	77	6.7	333	5.3	1,174	2.4
Sex	39	3.4	226	3.6	460	0.9
Age - any	15	1.3	72	1.1	169	0.3
Age	15	1.3	73	1.2	218	0.4
Date of birth	15	1.3	72	1.1	169	0.3
Country of birth	780	67.5	4,661	74.0	32,027	65.1
Civil status	906	78.4	5,440	86.4	37,687	76.7
Profession	755	65.4	4,513	71.7	18,790	38.2
Profession	777	67.3	4,620	73.4	19,740	40.2
Profession category	985	85.3	5,548	88.1	27,725	56.4
Vaccination*						
Type of first vaccine	740	84.2	3,725	77.8	21,461	53.5
Date of first vaccine	13	11.8	62	8.2	568	5.1
Type of second vaccine	54	49.1	368	48.4	4,315	38.5
Date of second vaccine	10	17.2	40	9.9	249	4.3
